# Mechanical Ventilation Drives Inflammation in Severe Viral Bronchiolitis

**DOI:** 10.1371/journal.pone.0083035

**Published:** 2013-12-11

**Authors:** Marije P. Hennus, Adrianus J. van Vught, Mark Brabander, Frank Brus, Nicolaas J. Jansen, Louis J. Bont

**Affiliations:** 1 Department of Paediatric Intensive Care, Wilhelmina Children’s Hospital / University Medical Center Utrecht, Utrecht, The Netherlands; 2 Department of Paediatrics, Haga Hospital/Location Juliana Children’s Hospital, The Hague, The Netherlands; 3 Department of Paediatric Infectious Diseases, Wilhelmina Children’s Hospital/University Medical Center Utrecht, Utrecht, The Netherlands; University of Tennessee Health Science Center, United States of America

## Abstract

**Introduction:**

Respiratory insufficiency due to severe respiratory syncytial virus (RSV) infection is the most frequent cause of paediatric intensive care unit admission in infants during the winter season. Previous studies have shown increased levels of inflammatory mediators in airways of mechanically ventilated children compared to spontaneous breathing children with viral bronchiolitis. In this prospective observational multi-center study we aimed to investigate whether this increase was related to disease severity or caused by mechanical ventilation.

**Materials and Methods:**

Nasopharyngeal aspirates were collected <1 hour before intubation and 24 hours later in RSV bronchiolitis patients with respiratory failure (n = 18) and non-ventilated RSV bronchiolitis controls (n = 18). Concentrations of the following cytokines were measured: interleukin (IL)-1α, IL-1β, IL-6, monocyte chemotactic protein (MCP)-1 and macrophage inflammatory protein (MIP)-1α.

**Results:**

Baseline cytokine levels were comparable between ventilated and non-ventilated infants. After 24 hours of mechanical ventilation mean cytokine levels, except for MIP-1α, were elevated compared to non-ventilated infected controls: IL-1α (159 versus 4 pg/ml, p<0.01), IL-1β (1068 versus 99 pg/ml, p<0.01), IL-6 (2343 versus 958 pg/ml, p<0.05) and MCP-1 (174 versus 26 pg/ml, p<0.05).

**Conclusions:**

Using pre- and post-intubation observations, this study suggests that endotracheal intubation and subsequent mechanical ventilation cause a robust pulmonary inflammation in infants with RSV bronchiolitis.

## Introduction

Respiratory syncytial virus (RSV) is the most common viral cause of seasonal acute respiratory tract illness in infants worldwide. The clinical manifestations range from mild upper respiratory tract symptoms (cough, coryza, rhinorrhea and conjunctivitis), to severe lower respiratory tract infection (LRTI) and even life-threatening respiratory insufficiency requiring mechanical ventilation. Treatment for RSV LRTI is largely supportive and no effective vaccine is currently available [1]. More than 50% of all infants are infected with RSV during the first year of life and at age 2 almost all children have been infected [2]. About 1% to 2% of all children will need hospitalization and about 10% of these hospitalized children, approximately 0.1% of all children, will require mechanical ventilation for a severe RSV LRTI during the first year of life [3]. Accordingly, RSV LRTI is the most frequent cause of non-elective paediatric intensive care unit (PICU) admission for mechanical ventilatory support in infants during the winter season [4].

RSV infection is associated with the production and release of large amounts of proinflammatory cytokines and chemokines [5], [6]. Several studies have demonstrated a correlation between disease severity with both local immune response [7]–[10] and viral load [11]–[14]. When comparing ventilated with non-ventilated infected infants, an association between the inflammatory response and disease severity was also found [14]–[17]. In these studies however, all samples from ventilated infected infants were collected after initiation of mechanical ventilation. Subsequently, observed cytokine concentrations might not only reflect virus-induced pulmonary inflammation but also the immunological response to mechanical ventilation known as ventilator induced lung injury [18].

We sought to investigate to what extent increased local inflammation in mechanically ventilated children with RSV bronchiolitis resulted from disease severity or endotracheal intubation and initial mechanical ventilation. We hypothesized that differences in cytokine levels between mechanically ventilated and non-ventilated RSV infected infants were absent at time of intubation but developed during the early phase of mechanical ventilation. To test this hypothesis we studied local cytokine levels prior to endotracheal intubation and again 24 hours later.

## Materials and Methods

The study was approved by the regional Medical Ethical Committee South West Holland and the Ethics Review Committee of the University Medical Center Utrecht and conducted according to the principles expressed in the Declaration of Helsinki. All parents provided written, informed consent.

### Selection of patients

Infants less than 13 months old with a proven RSV infection and respiratory insufficiency requiring mechanical ventilation were enrolled in the Wilhelmina’s Children’s Hospital, Utrecht, the Netherlands during 2 winter epidemics (2009–2011). The decision to intubate and start mechanical ventilation was at the discretion of the attending physician. During the same period, hospitalized children less than 13 months with proven RSV LRTI without the need for mechanical ventilation were enrolled as non-ventilated controls in two hospitals in the Netherlands (Wilhelmina’s Children’s Hospital, Utrecht and Juliana Children's Hospital/Haga Teaching Hospital, The Hague). Infants with underlying chronic lung disease, cyanotic congenital heart disease, Down’s syndrome and/or prematurity (gestational age < 37 weeks) were excluded.

### Collection of materials

For this study we measured cytokine levels in nasopharyngeal aspirates (NPA). A strong correlation exists between cytokine concentrations determined in upper and lower airways [9], [15], [17], [19] In order to collect material before the onset of mechanical ventilation as well as to optimize the overall sample collection rate, undiluted NPAs were collected shortly (< 1 hour) before intubation. A second NPA was collected 24 hours after intubation. In non-ventilated patients, a NPA sample was taken on admission and 24 hours later. Aspirates were placed on ice immediately and stored at –80°C for later cytokine analysis.

### Measurements

Interleukin (IL)-1α, IL-1β, IL-6, monocyte chemotactic protein (MCP)-1 and macrophage inflammatory protein (MIP)-1α were measured in NPAs by enzyme-linked immunosorbent assay according to manufacturer's instructions (R&D Systems, United Kingdom). The lower detection limit for IL-1α was 8 pg/ml; for IL-1β 70 pg/ml, for IL-6 32 pg/ml, for MCP-1 32 pg/ml and for MIP-1α 80 pg/ml. When cytokines were not detectable, the minimum detectable level divided by 2 was used in the calculations. RSV infection was diagnosed by PCR as described previously [14]. RSV concentrations were analysed using real-time PCR. Low cycle time (CT) values indicate high RSV concentrations, while high CT values (with a maximum of 40 cycles) represent low viral loads.

### Statistical analysis

All data are expressed as mean (±SD). To detect differences between groups a paired t-Test was used and a p-value of less than 0.05 was considered statistically significant. Correlation between viral load and cytokine concentration was analysed with Spearman's rank correlation coefficient. Statistical analyses were carried out using GraphPad PRISM 5 (La Jolla, United States of America).

## Results

### Patient Characteristics

**Figure 1:**
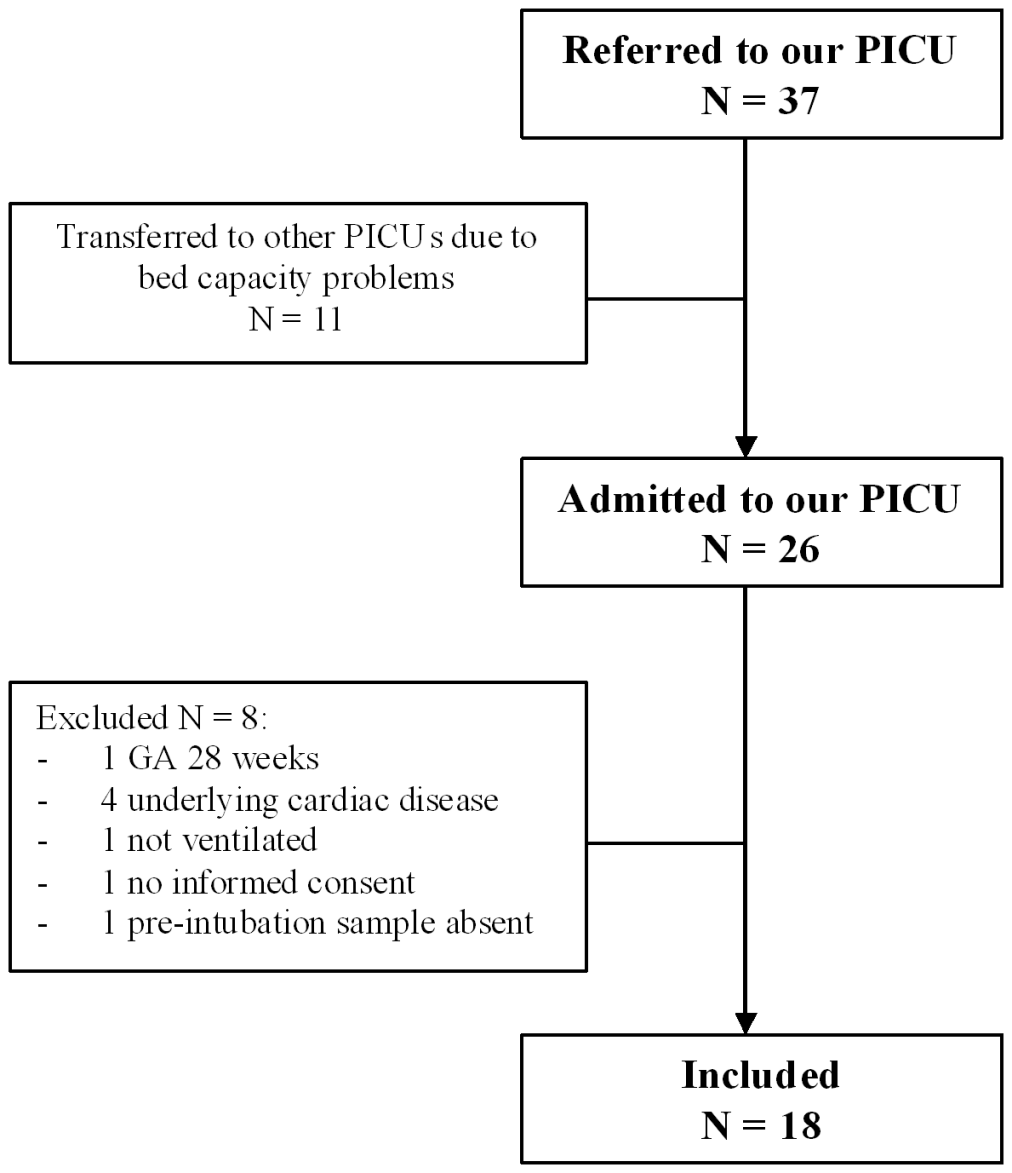
Flow chart of RSV-infected patients referred to our PICU for ventilatory support and inclusion. PICU: paediatric intensive care unit, GA: gestational age.

Characteristics of patients and controls are shown in table 1. During the study period 37 RSV-infected patients were referred to our PICU for ventilatory support resulting in inclusion of 18 patients (figure 1). At time of sampling, six patients in this study group had positive bacterial cultures from tracheal aspirates (*Moraxella catarrhalis* n = 2, *Haemophilus influenza* n = 4, *Streptococcus pneumoniae* n = 2, *Staphylococcus aureus* n = 3, *Streptococcus pyogenes* n = 1) whereas 3 others had a viral co-infection (*Rhinovirus* n = 3, *Adenovirus* n = 1). None of the patients received ribavarin or systemic steroids. All patients survived.

**Table 1:** Patient characteristics of spontaneous breathing and mechanically ventilated RSV-infected infants.

	Spontaneous Breathing	Mechanically Ventilated	p-value
Nr patients included	18	18	NS
Male patients, nr (%)	11 (61)	11 (61)	NS
Age in days, mean (SD)	95 (57)	72 (90)	0,04
Weight in kg, mean (SD)	6,1 (5)	4,7 (1,4)	0,02
Days ill at t = 0, mean (SD)	2,8 (3)	3,7 (2)	0,11
Viral co-infections, nr (%)	-	3 (17)	
Bacterial pneumonia, nr (%)	-	6 (33)	
**T = 0 hours**			
Blood gas analysis, nr (%)	5 (28)	12 (67)	
pH/PaCO2/BE/Bic, mean (SD)	7,39(0,04)/53,7(12)/3(4)/28,6(3)	7,19(0,08)/72,4(22)/1,8(2)/25,9(8)	
**T = 24 hours**			
Vt in ml/ kg, mean (SD)	-	7,6 (2)	
Peak ventilator pressure in cm H2O, mean (SD)	-	27,5 (5)	
Ventilator FiO2, mean (SD)	-	0,37 (0,05)	
Transcutaneous saturation, mean (SD)	98 (1)	98 (2)	
Blood gas analysis, nr (%)	1 (6)	18 (100)	
pH/PaCO2/BE/Bic, mean (SD)	7,32(0)/ 65,0(0)/8,0(0)/33,8(0)	7,38(0,05)/45,3(7)/2,2(3)/27,2(3)	

Nr: number of patients; kg: kilograms; T = 0 for ventilated patients is < 1 hour before intubation, for non-ventilated patients on admission; T = 24 is 24 hours after the first sample in both groups; NS: not significant; PaCO_2_: arterial carbon dioxide tension; BE: base excess; Bic: bicarbonate; FiO_2_: fraction of inspired oxygen; Vt: Tidal volume.

The control subjects were included in the Wilhelmina Children’s Hospital (n = 16) and in the Juliana Children's Hospital/Haga Teaching Hospital (n = 2). As expected, mechanically ventilated infants were significantly younger and weighed less than their spontaneous breathing controls (table 1).

### Cytokine levels at baseline are comparable for ventilated and non-ventilated infected infants

**Figure 2:**
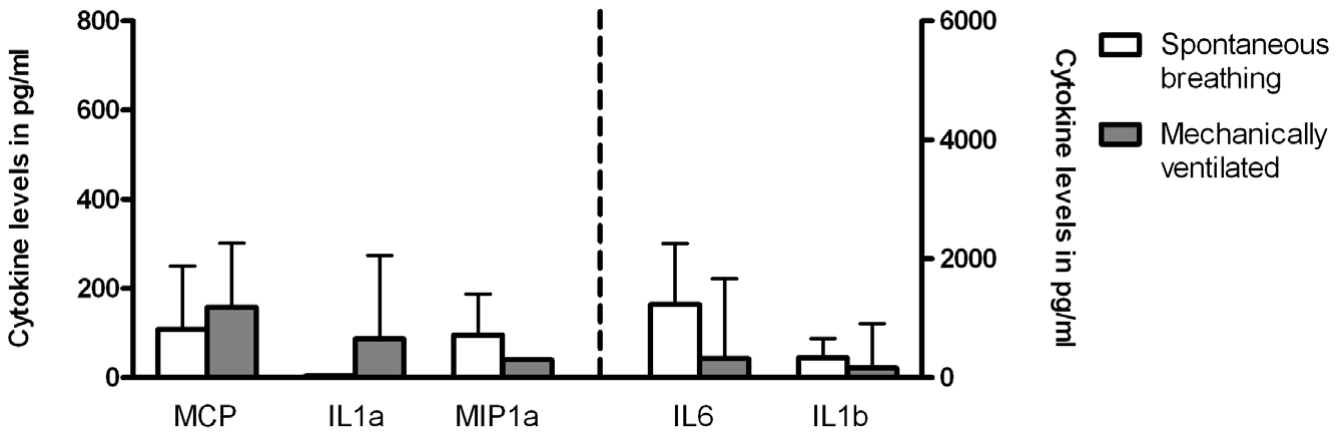
Nasopharyngeal aspirate cytokine levels at baseline. An undiluted nasopharyngeal aspirate was taken from respiratory insufficient RSV-infected infants (age < 13 months) <1 hour before intubation. Aspirates of RSV-infected children (age < 13 months) without the need of mechanical ventilation were collected on admission. The graph shows cytokines levels for spontaneous breathing infants (white bars) and for respiratory insufficient infants just before mechanical ventilation (grey bars). IL-1α: interleukin-1α; IL-1β: interleukin-1β; IL-6: interleukin-6; MCP-1: monocyte chemotactic protein; MIP-1α: macrophage inflammatory protein-1α. Bars represent mean ± sd (n =  11 infants for IL1a MV only; all other groups n = 18 infants). No differences were found between the two groups using a paired t-test.

RSV-induced pulmonary inflammation at baseline, reflected by NPA IL-1α, IL-1β, IL-6, MCP-1 and MIP-1α concentrations, did not differ between ventilated and non-ventilated patients (figure 2).

### Mechanical ventilation enhances pulmonary inflammation

**Figure 3:**
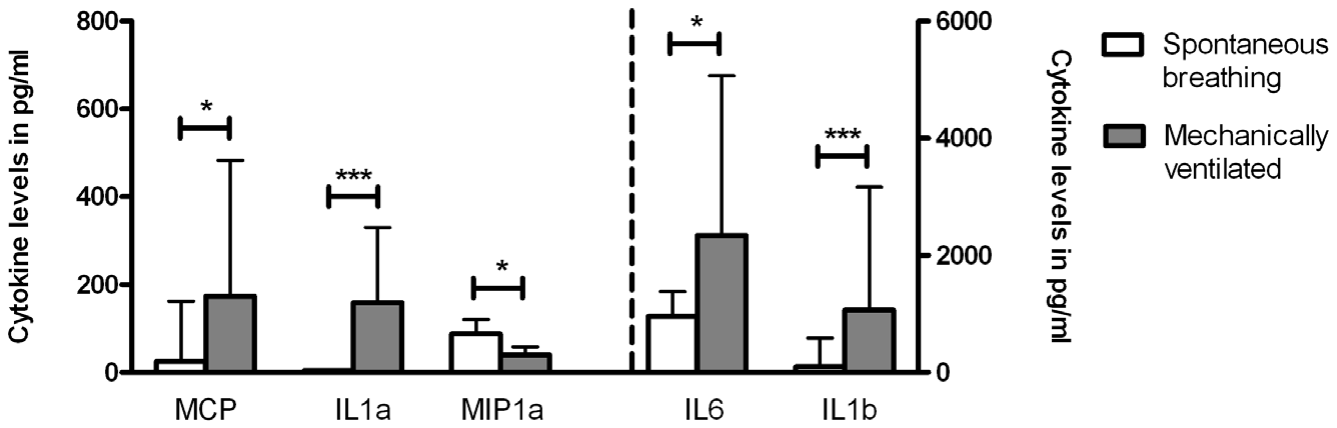
Nasopharyngeal aspirate cytokine levels after 24 hours. An undiluted nasopharyngeal aspirate was taken 24-infected infants (age < 13 months). The graph shows cytokines levels for both spontaneous breathing (white bars) and mechanically ventilated infants (grey bars). IL-1α: interleukin-1α; IL-1β: interleukin-1β; IL-6: interleukin-6; MCP-1: monocyte chemotactic protein; MIP-1α: macrophage inflammatory protein-1α. Bars represent mean ± sd (n =  11 infants for IL1a MV only; all other groups n = 18 infants). A paired t-test was used to compare differences between the two groups (* =  P<0.05, ** =  P<0.01, *** =  P<0.001).

After 24 hours all pro-inflammatory cytokine concentrations, except for MIP-1α, were significantly enhanced in the mechanically ventilated group as compared to the non-ventilated controls (figure 3). In the spontaneous breathing group, all mean cytokine concentrations at t = 24 hours showed a decreasing trend compared to baseline values (IL-1β 430 vs. 382 pg/ml, for IL-6 1571 vs. 1037 pg/ml, for MCP-1 184 vs. 153 pg/ml and for MIP-1α 177 vs. 90 pg/ml), except for IL-1α (59 vs. 80 pg/ml). For the ventilated patients, all mean cytokine concentrations clearly increased during 24 hours of mechanical ventilation (IL-1α 87 vs. 159 pg/ml; for IL-1β 160 vs. 1068 pg/ml, for IL-6 322 vs. 2342 pg/ml and for MCP-1 158 vs. 174 pg/ml), except for MIP-1α (40 vs. 40 pg/ml). These results suggest that mechanical ventilation during RSV infection clearly enhances pulmonary inflammation as compared to RSV infection alone reflected by NPA pro-inflammatory cytokines.

### Viral loads

Although nasopharyngeal viral loads taken on t = 0 in ventilated infants were higher than loads measured in the spontaneous breathing group (CT 20.6 vs. 22.9), this difference did not reach statistical significance (p = 0.06). There was no correlation found between viral loads and cytokine levels.

## Discussion

This is the first study investigating cytokine levels before and after endotracheal intubation for RSV bronchiolitis. We studied to what extent intubation and subsequent mechanical ventilation in infants with RSV bronchiolitis could have impacted on pulmonary inflammation in comparison with infants with RSV bronchiolitis who were not mechanically ventilated. We found that the need for mechanical ventilation in RSV-infected infants was not associated with enhanced virus-induced pulmonary inflammation at baseline. Endotracheal intubation and subsequent mechanical ventilation induced additional inflammation expressed by increased levels of NPA cytokine concentrations after 24 hours.

The association between immunological response and disease severity in spontaneous breathing versus mechanically ventilated RSV infected children has been previously studied by our group and others [14]–[16]. In these studies however cytokine analysis was performed on samples collected while patients were already ventilated. Therefore, observed cytokine concentrations might not only reflect viral induced pulmonary inflammation but also the immunological response to intubation and mechanical ventilation which complicates interpretation of these results. However, we now show that virus induced pulmonary inflammation in infants with mild RSV disease (spontaneous breathing group) was similar to what was observed in infants with severe RSV disease (ventilated group) at time of intubation. Hence, the need for mechanical ventilation in previously healthy infants was not explained by enhanced viral induced pulmonary inflammation or viral load. Most likely, other patient-related factors, such as age and/or weight [20] and genetic susceptibility [16] played an important role in the development of life-threatening RSV bronchiolitis.

The increase in cytokine levels in infants with respiratory failure due to RSV bronchiolitis was attributable to intubation and mechanical ventilation, despite the use of mean tidal volumes of 7.6 ml/kg, reflecting a so-called lung protective ventilatory strategy [21]–[24]. Furthermore, disease duration at presentation did not differ between the 2 groups but might be attributable to the small number of patients studied. Subsequently, we compared the 2 groups at similar time points: baseline sample of ventilated children taken after 3.7 days of disease versus 24 hour sample of spontaneous breathing children taken after 3.8 days of disease. No differences were found, except for IL-1α (data not shown). In addition, virus induced inflammation did not increase during the first 24 hours in spontaneous breathing controls. This strengthens the observation that enhanced inflammation is indeed attributable to ventilator induced inflammation. These findings corroborate experimental studies demonstrating that mechanical ventilation augments pulmonary inflammation during viral infection [25], [26]. As we have previously shown, the current study provides further support that inflammation in ventilated children is attributable to a ventilator induced molecular stress response superimposed on virus-induced innate immunity activation with little interaction [26].

The current study adds to our knowledge of the inflammatory consequences of endotracheal intubation and initial ventilation: a robust inflammatory response is superimposed on top of existent viral induced inflammation. Furthermore it underscores the need to adhere to lung protective ventilation strategies in patients with severe respiratory infections. More importantly, one could argue that given the observed additional inflammatory hit resulting from invasive mechanical ventilation, non-invasive ventilatory strategies may be considered for infants with life-threatening RSV bronchiolitis to prevent further airway inflammation.

This study has several strengths. Firstly, even in healthy children mechanical ventilation elicits an inflammatory response within hours [27]. Therefore, a baseline sample just prior to intubation, as was collected in our study, was essential. Secondly, the observed enhanced inflammatory response during mechanical ventilation was observed while using a lung protective mode of mechanical ventilation. On the other hand, we have to consider the following limitations. Firstly, mechanically ventilated infants were significantly younger than their spontaneous breathing controls. However, we have previously shown that neonatal innate immune responses in whole blood are biased against the production of pro-inflammatory cytokinesis at birth and this production gradually increases during the first month of life [28]. No studies have investigated age dependent cytokinesis production in nasal specimens. However, in light of fetomaternal tolerance, it seems likely that neonatal production of proinflammatory cytokines is generally impaired [29]. Therefore, the pro-inflammatory response seen in our ventilated group cannot be explained by their younger age. Secondly, a healthy age-matched control group requiring invasive mechanical ventilation for at least 24 hours was lacking for practical reasons. This group however could have expanded our knowledge on the immunological effects of invasive mechanical ventilation in normal lungs and on the effects of stress, pain and sedation in intubated infants. Thirdly, a RSV-infected control group matched for age and disease severity not exposed to invasive mechanical ventilation would be desirable. Unfortunately, this was not feasible during the study period as non-invasive mechanical ventilation was not yet available in our paediatric intensive care unit. Finally, we did not distinguish viral co-infection because these were uncommon (n = 3). Bacterial pulmonary co-infections are common in severe RSV-infections requiring PICU admission [30] and might also influence pulmonary inflammation. Six of our ventilated patients (33%) were pragmatically diagnosed with concurrent bacterial pneumonia. Cytokine concentrations of these 6 patients however did not differ from ventilated patients without concurrent bacterial pneumonia (data not shown).

In summary, we provide the first evidence that the need for mechanical ventilation in RSV-infected infants is not preceded by enhanced viral induced pulmonary inflammation. We showed that invasive mechanical ventilation is likely to aggravate pulmonary inflammation, warranting further development of lung-protective non-invasive ventilatory support for this group of patients.

## References

[1] Wright M, Piedimonte G (2011) Respiratory syncytial virus prevention and therapy: past, present, and future. Pediatr Pulmonol 46: 324–347. 21438168 10.1002/ppul.21377

[2] American Academy of Pediatrics Subcommittee on Diagnosis and Management of Bronchiolitis (2006) Diagnosis and management of bronchiolitis. Pediatrics 118: 1774–1793. 17015575 10.1542/peds.2006-2223

[3] Behrendt CE, Decker MD, Burch DJ, Watson PH (1998) International variation in the management of infants hospitalized with respiratory syncytial virus. International RSV Study Group. Eur J Pediatr 157: 215–220. 9537488 10.1007/s004310050798

[4] Simoes EA (1999) Respiratory syncytial virus infection. Lancet 354: 847–852. 10485741 10.1016/S0140-6736(99)80040-3

[5] Hornsleth A, Loland L, Larsen LB (2001) Cytokines and chemokines in respiratory secretion and severity of disease in infants with respiratory syncytial virus (RSV) infection. J Clin Virol 21: 163–170. 11378497 10.1016/s1386-6532(01)00159-7

[6] McNamara PS, Ritson P, Selby A, Hart CA, Smyth RL (2003) Bronchoalveolar lavage cellularity in infants with severe respiratory syncytial virus bronchiolitis. Arch Dis Child 88: 922–926. 14500316 10.1136/adc.88.10.922 PMC1719332

[7] Bennett BL, Garofalo RP, Cron SG, Hosakote YM, Atmar RL, et al (2007) Immunopathogenesis of respiratory syncytial virus bronchiolitis. J Infect Dis 195: 1532–1540. 17436234 10.1086/515575

[8] Garcia C, Soriano-Fallas A, Lozano J, Leos N, Gomez AM, et al (2012) Decreased innate immune cytokine responses correlate with disease severity in children with respiratory syncytial virus and human rhinovirus bronchiolitis. Pediatr Infect Dis J 31: 86–89. 21829141 10.1097/INF.0b013e31822dc8c1

[9] Garofalo RP, Patti J, Hintz KA, Hill V, Ogra PL, et al (2001) Macrophage inflammatory protein-1alpha (not T helper type 2 cytokines) is associated with severe forms of respiratory syncytial virus bronchiolitis. J Infect Dis 184: 393–399. 11471095 10.1086/322788

[10] Welliver TP, Garofalo RP, Hosakote Y, Hintz KH, Avendano L, et al (2007) Severe human lower respiratory tract illness caused by respiratory syncytial virus and influenza virus is characterized by the absence of pulmonary cytotoxic lymphocyte responses. J Infect Dis 195: 1126–1136. 17357048 10.1086/512615 PMC7109876

[11] Buckingham SC, Bush AJ, DeVincenzo JP (2000) Nasal quantity of respiratory syncytical virus correlates with disease severity in hospitalized infants. Pediatr Infect Dis J 19: 113–117. 10693996 10.1097/00006454-200002000-00006

[12] El Saleeby CM, Bush AJ, Harrison LM, Aitken JA, DeVincenzo JP (2011) Respiratory syncytial virus load, viral dynamics, and disease severity in previously healthy naturally infected children. J Infect Dis 204: 996–1002. 21881113 10.1093/infdis/jir494 PMC3203391

[13] Fodha I, Vabret A, Ghedira L, Seboui H, Chouchane S, et al (2007) Respiratory syncytial virus infections in hospitalized infants: association between viral load, virus subgroup, and disease severity. J Med Virol 79: 1951–1958. 17935185 10.1002/jmv.21026

[14] Houben ML, Coenjaerts FE, Rossen JW, Belderbos ME, Hofland RW, et al (2010) Disease severity and viral load are correlated in infants with primary respiratory syncytial virus infection in the community. J Med Virol 82: 1266–1271. 20513094 10.1002/jmv.21771 PMC7167003

[15] Bont L, Heijnen CJ, Kavelaars A, van Aalderen WM, Brus F, et al (2001) Local interferon-gamma levels during respiratory syncytial virus lower respiratory tract infection are associated with disease severity. J Infect Dis 184: 355–358. 11443563 10.1086/322035

[16] Faber TE, Schuurhof A, Vonk A, Koppelman GH, Hennus MP, et al (2012) IL1RL1 gene variants and nasopharyngeal IL1RL-a levels are associated with severe RSV bronchiolitis: a multicenter cohort study. PLoS One 7: e34364. 22574108 10.1371/journal.pone.0034364 PMC3344820

[17] Sheeran P, Jafri H, Carubelli C, Saavedra J, Johnson C, et al (1999) Elevated cytokine concentrations in the nasopharyngeal and tracheal secretions of children with respiratory syncytial virus disease. Pediatr Infect Dis J 18: 115–122. 10048682 10.1097/00006454-199902000-00007

[18] Tremblay LN, Slutsky AS (2006) Ventilator-induced lung injury: from the bench to the bedside. Intensive Care Med 32: 24–33. 16231069 10.1007/s00134-005-2817-8

[19] Joshi P, Kakakios A, Jayasekera J, Isaacs D (1998) A comparison of IL-2 levels in nasopharyngeal and endotracheal aspirates of babies with respiratory syncytial viral bronchiolitis. J Allergy Clin Immunol 102: 618–620. 9802370 10.1016/s0091-6749(98)70278-7

[20] Soilly AL, Ferdynus C, Desplanches O, Grimaldi M, Gouyon JB (2012) Paediatric intensive care admissions for respiratory syncytial virus bronchiolitis in France: results of a retrospective survey and evaluation of the validity of a medical information system programme. Epidemiol Infect 140: 608–616. 21733254 10.1017/S0950268811001208

[21] Amato MB, Barbas CS, Medeiros DM, Magaldi RB, Schettino GP, et al (1998) Effect of a protective-ventilation strategy on mortality in the acute respiratory distress syndrome. N Engl J Med 338: 347–354. 9449727 10.1056/NEJM199802053380602

[22] Chiumello D, Pristine G, Slutsky AS (1999) Mechanical ventilation affects local and systemic cytokines in an animal model of acute respiratory distress syndrome. Am J Respir Crit Care Med 160: 109–116. 10390387 10.1164/ajrccm.160.1.9803046

[23] The Acute Respiratory Distress Syndrome Network (2000) Ventilation with lower tidal volumes as compared with traditional tidal volumes for acute lung injury and the acute respiratory distress syndrome. The Acute Respiratory Distress Syndrome Network. N Engl J Med 342: 1301–1308. 10793162 10.1056/NEJM200005043421801

[24] Zosky GR, Cannizzaro V, Hantos Z, Sly PD (2009) Protective mechanical ventilation does not exacerbate lung function impairment or lung inflammation following influenza A infection. J Appl Physiol 107: 1472–1478. 19745194 10.1152/japplphysiol.00393.2009

[25] Bem RA, van Woensel JB, Bos AP, Koski A, Farnand AW, et al (2009) Mechanical ventilation enhances lung inflammation and caspase activity in a model of mouse pneumovirus infection. Am J Physiol Lung Cell Mol Physiol 296: L46–L56. 18996903 10.1152/ajplung.00467.2007 PMC2636950

[26] Hennus MP, Janssen R, Pennings JL, Hodemaekers HM, Kruijsen D, et al (2012) Host response to mechanical ventilation for viral respiratory tract infection. Eur Respir J 40: 1508–1515. 22496321 10.1183/09031936.00177111

[27] Plotz FB, Vreugdenhil HA, Slutsky AS, Zijlstra J, Heijnen CJ, et al (2002) Mechanical ventilation alters the immune response in children without lung pathology. Intensive Care Med 28: 486–492. 11967605 10.1007/s00134-002-1216-7 PMC7095146

[28] Belderbos ME, van Bleek GM, Levy O, et al (2009) Skewed pattern of Toll-like receptor 4-mediated cytokine production in human neonatal blood: low LPS-induced IL-12p70 and high IL-10 persist throughout the first month of life. Clin Immunol 133: 228–237. 19648060 10.1016/j.clim.2009.07.003 PMC2892115

[29] Levy O (2007) Innate immunity of the newborn: basic mechanisms and clinical correlates. Nat Rev Immunol 7: 379–390. 17457344 10.1038/nri2075

[30] Thorburn K, Harigopal S, Reddy V, Taylor N, van Saene HK (2006) High incidence of pulmonary bacterial co-infection in children with severe respiratory syncytial virus (RSV) bronchiolitis. Thorax 61: 611–615. 16537670 10.1136/thx.2005.048397 PMC2104657

